# Two Case Reports on Thalamic and Basal Ganglia Involvement in Children with Dengue Fever

**DOI:** 10.1155/2016/7961368

**Published:** 2016-07-11

**Authors:** Guwani Liyanage, Lihini Adhikari, Saraji Wijesekera, Maheshaka Wijayawardena, Suchithra Chandrasiri

**Affiliations:** ^1^Department of Paediatrics, Faculty of Medical Sciences, University of Sri Jayewardenepura, Sri Soratha Mawatha, 10250 Nugegoda, Sri Lanka; ^2^Professorial Paediatric Unit, Colombo South Teaching Hospital, Kalubowila, 10350 Dehiwala, Sri Lanka

## Abstract

There have been increasing numbers of case reports of dengue infection with unusual manifestations. Such unusual manifestations including acute liver failure and encephalopathy could be manifested even in the absence of significant plasma leakage. Further, severe organ involvement including nervous system involvement indicates severe dengue infection. However, neurological manifestations of dengue fever are rare. This is the first case report of dengue infection with thalamic and basal ganglia involvement in Sri Lanka.

## 1. Introduction

The clinical manifestations of dengue fever form a broad spectrum including uncomplicated dengue fever, dengue hemorrhagic fever, and dengue shock syndrome according to the traditional classification. However, in 2009, World health Organization (WHO) has introduced a new classification and it categorizes the disease into dengue without warning signs, dengue with warning signs, and severe dengue [1]. Importantly, encephalopathy, encephalitis, and any other unusual neurological involvement are recognized as manifestations of sever dengue. However, neurological manifestations are rare in dengue infection. There are few case reports of thalamic and basal ganglia involvement in dengue fever [2–4]. Most of these patients had full recovery with transient thalamic involvement on neuroimaging. However, this is the first report of dengue infection complicated by thalamic and basal ganglia involvement in children in Sri Lanka.

## 2. Case Reports

Patient 1 was a 10-year-old girl admitted with 2 days of fever, retroorbital headache, vomiting, and myalgia. Her white blood cell (WBC) count on the 3rd day was 2.2 × 10^3^ with 65% neutrophils and platelet count of 117,000. Alanine transaminase (ALT) was 17.8 and aspartate transaminase (AST) was 55.2. Treatment was commenced as dengue fever in febrile phase. By the 4th day, she became sleepy and lethargic and the level of consciousness gradually deteriorated. She was moved to high dependency care for further management. On neurological examination, she had increased tone in all four limbs with cogwheel rigidity. The lowest Glasgow coma scale documented was 10/15. Her vital parameters and serial packed cell volume readings remained stable without signs of fluid leakage.

**Figure 1:**
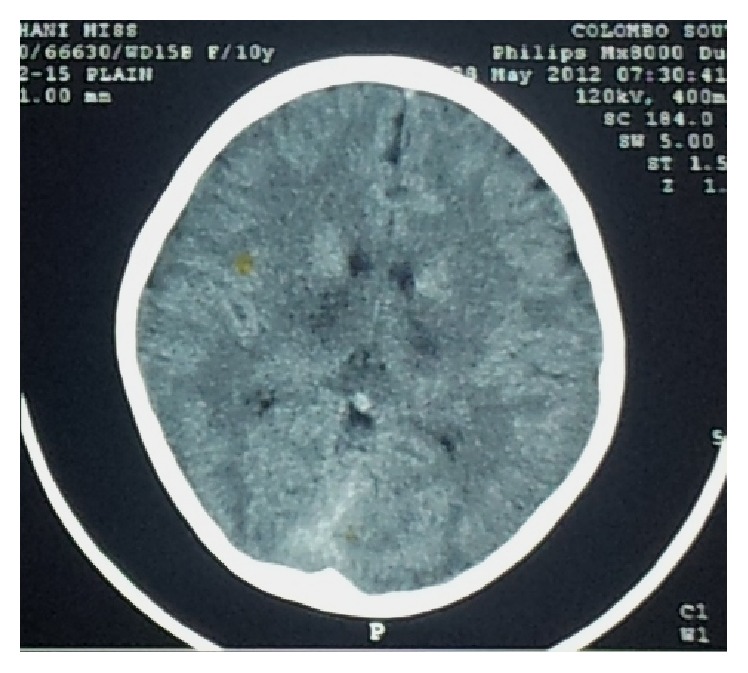
Axial brain CT image (with contrast) showing hypodense areas in the region of thalamus and basal ganglia.

Lowest WBC count (1.9 × 10^3^) was reported on day 5 of the illness and lowest platelet count was 50,000/mm^3^ on day 6. Liver enzymes deteriorated (ALT: 357, AST: 478) and serum albumin was 38 g/dL and INR was 1.17 on day 6. Tender liver was palpable 2 cm below the costal margin. Renal functions (sodium: 142 mEq/L, potassium: 4.2 mEq/L, blood urea: 7.6 mmol/L, and serum creatinine: 52 *μ*mol/L) remained normal. Serum calcium remained between 2.08 and 2.69 mmol/L. Primary dengue infection was suggested by the presence of IgM antibodies and absence of IgG antibodies to dengue virus. Dengue NS-1 antigen and the viral load were not performed due to lack of laboratory support. A CT scan revealed hypodense areas in bilateral thalamic and basal ganglia regions (Figure 1). Cerebrospinal fluid examination did not reveal any cells and had normal protein levels (42 mg/dL). Over the next 2 weeks following the acute illness, she showed complete recovery with no residual neurological disability.

Patient 2 was a 3-year-old girl who presented with 3 days of continuous high fever, intermittent abdominal pain, vomiting, and retroorbital headache. There had been one episode of gum bleeding on the first day of illness. She had been apparently a well child without any significant illnesses in the past. WBC count done on day 2, on request of the general practitioner, was 8.15 × 10^3^ with 85% neutrophils and platelet count was 120,000 mm^3^. She had right hypochondrial tenderness with just palpable liver on day 5. ALT and AST were 16 mEq/L and 69.7 mEq/L, respectively, on day 3 and they increased to 29.7 mEq/L and 122.2 mEq/L on day 5. Initial C-reactive protein was 6 mg/dL and remained normal. She was monitored for vital signs including urine output and packed cell volume (PCV). Baseline PCV was 32 and the highest noted was 35. The lowest platelet count documented was 67,000/mm^3^ and the lowest WBC count was 2.27 × 10^3^. She did not show signs of fluid leakage. However, there was a second episode of gum bleeding on day 5 with normal clotting profile (INR: 1.1 and APTT: 35). She was positive for IgM and negative for IgG antibodies for dengue virus.

**Figure 2:**
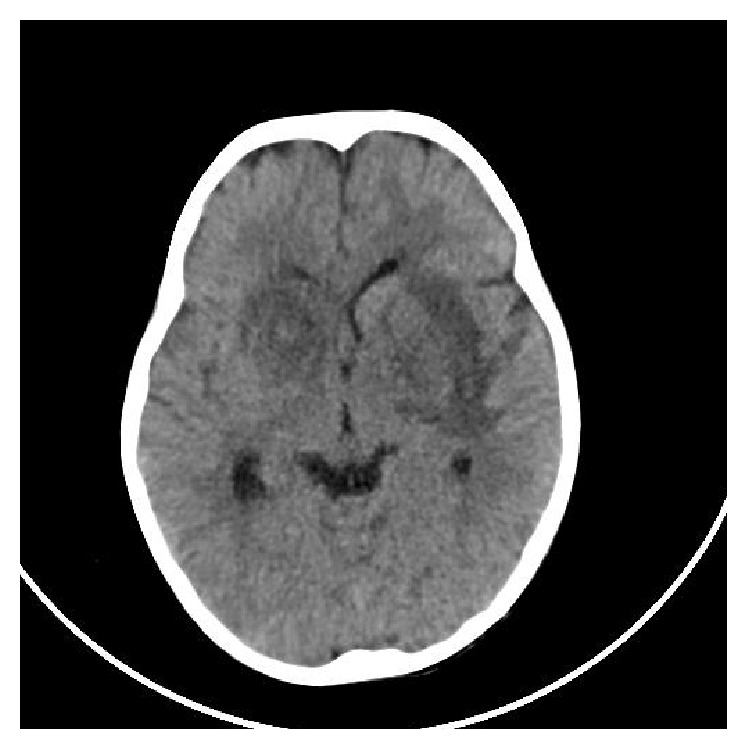
Axial brain CT image (noncontrast) of patient 2 showing hypodense areas in both thalami and basal ganglia.

On the 5th day of illness, she developed tremors in her upper limbs. She had cogwheel rigidity, mask like face, and staccato speech. Power was grade four in all four limbs with normal reflexes. CT scan of brain revealed diffuse hypodense areas in bilateral basal ganglia and thalamus with predominant involvement of right side (Figure 2). During the first 48 hours after the onset of neurological symptoms, she showed rapid deterioration and became aphasic with severe paucity of movements. Cerebrospinal fluid examination showed 2 lymphocytes without any polymorphs and normal proteins. She recovered dramatically and showed complete recovery within 3 weeks after onset of illness.

## 3. Discussion

There are increasing reports of dengue infection with unusual manifestations with brain involvement. The pathogenesis of neurological complications can be related to systemic effects of the infection, neurotropic effects of the virus, and the immune mediated effects [5]. Although such neurological complications have been reported among ages varying from 3 months to 60 years, the incidence is greater among children [6]. However, reports on basal ganglia and thalamic involvement are rare in children.

Mortality due to neurological complications is low and patients mostly die due to other multisystem involvement. Most patients recover completely by the time of discharge from the hospital. Imaging studies of patients with such neurological manifestations usually show only cerebral edema but rarely indicate encephalitis like alterations [7]. Hypodense areas seen in the regions of basal ganglia and thalami in our patients are probably due to inflammation.

However, both patients showed complete recovery indicating the transient nature of the thalamic and basal ganglia involvement. Supportive care and parent counseling are mainstay of management of these patients.

## Supplementary Material

Axial brain CT images showing hypodense areas in the region of thalamus and basal ganglia.
