# Supplementary material for: Two Case Reports on Thalamic and Basal Ganglia Involvement in Children with Dengue Fever
Source: Case Rep Infect Dis. 2016 Jul 11;2016:7961368. doi: 10.1155/2016/7961368 (PMC4958465; doi:10.1155/2016/7961368)

## Slide 1
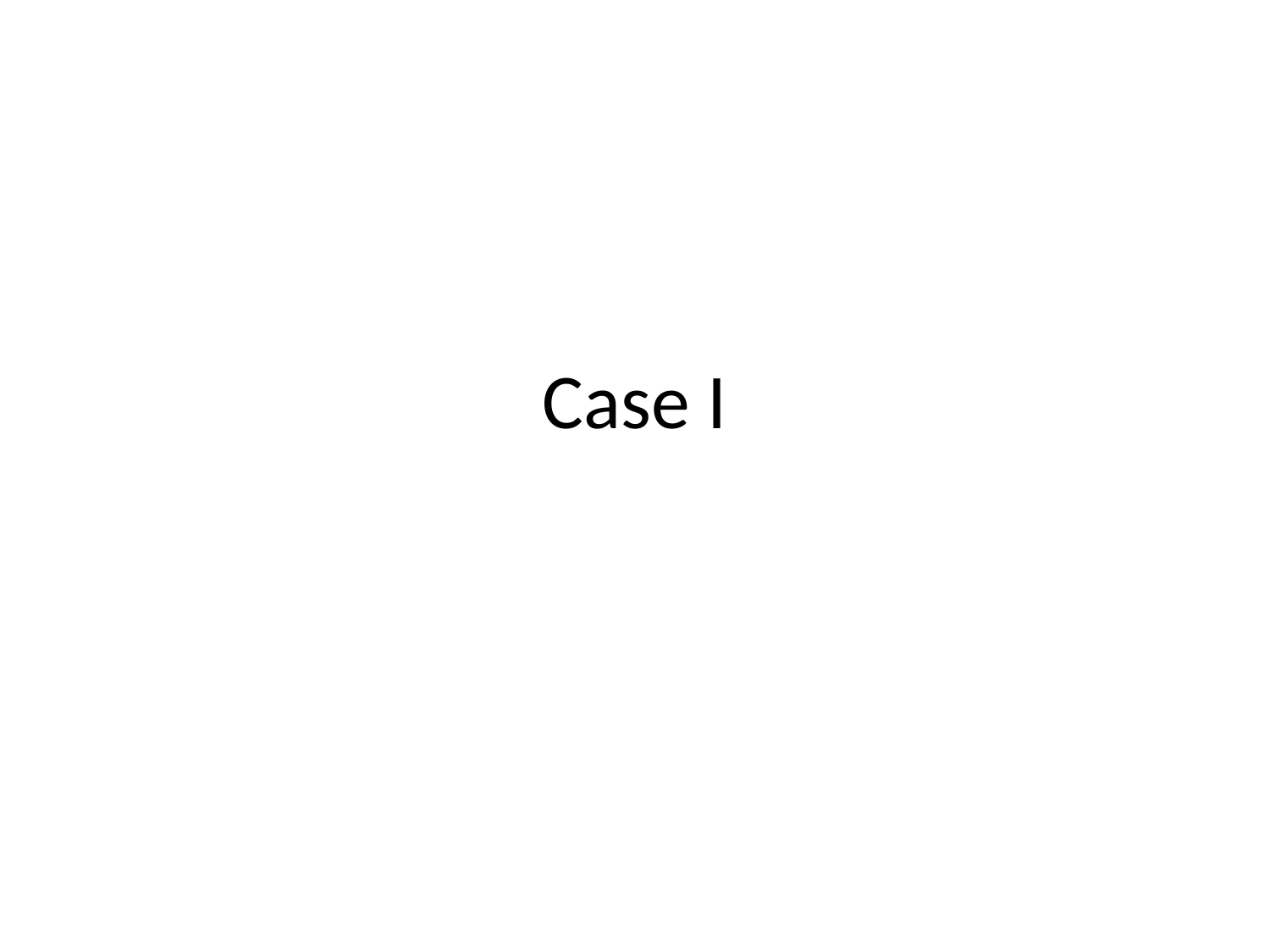

# Case I

## Slide 2
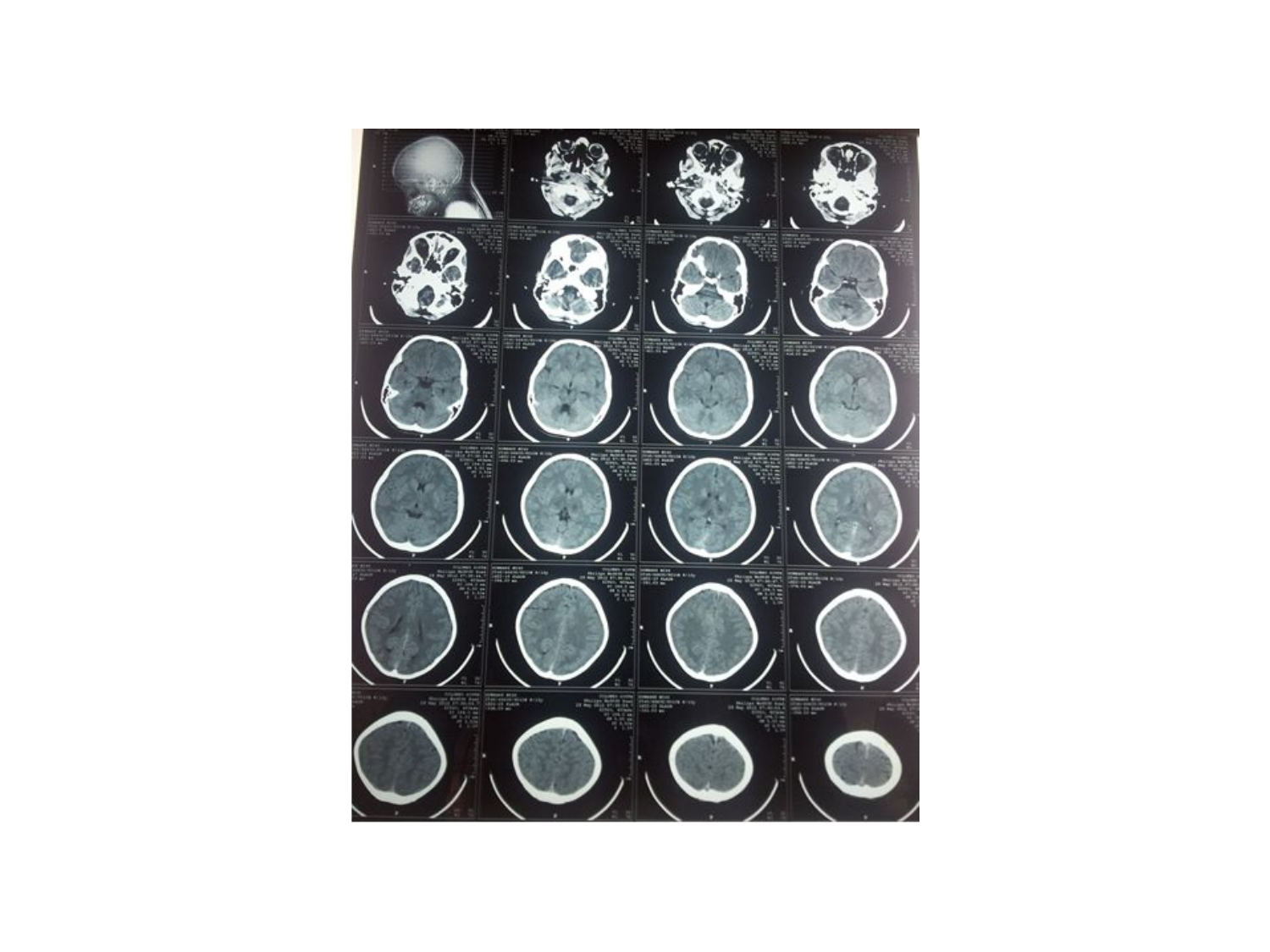

## Slide 3
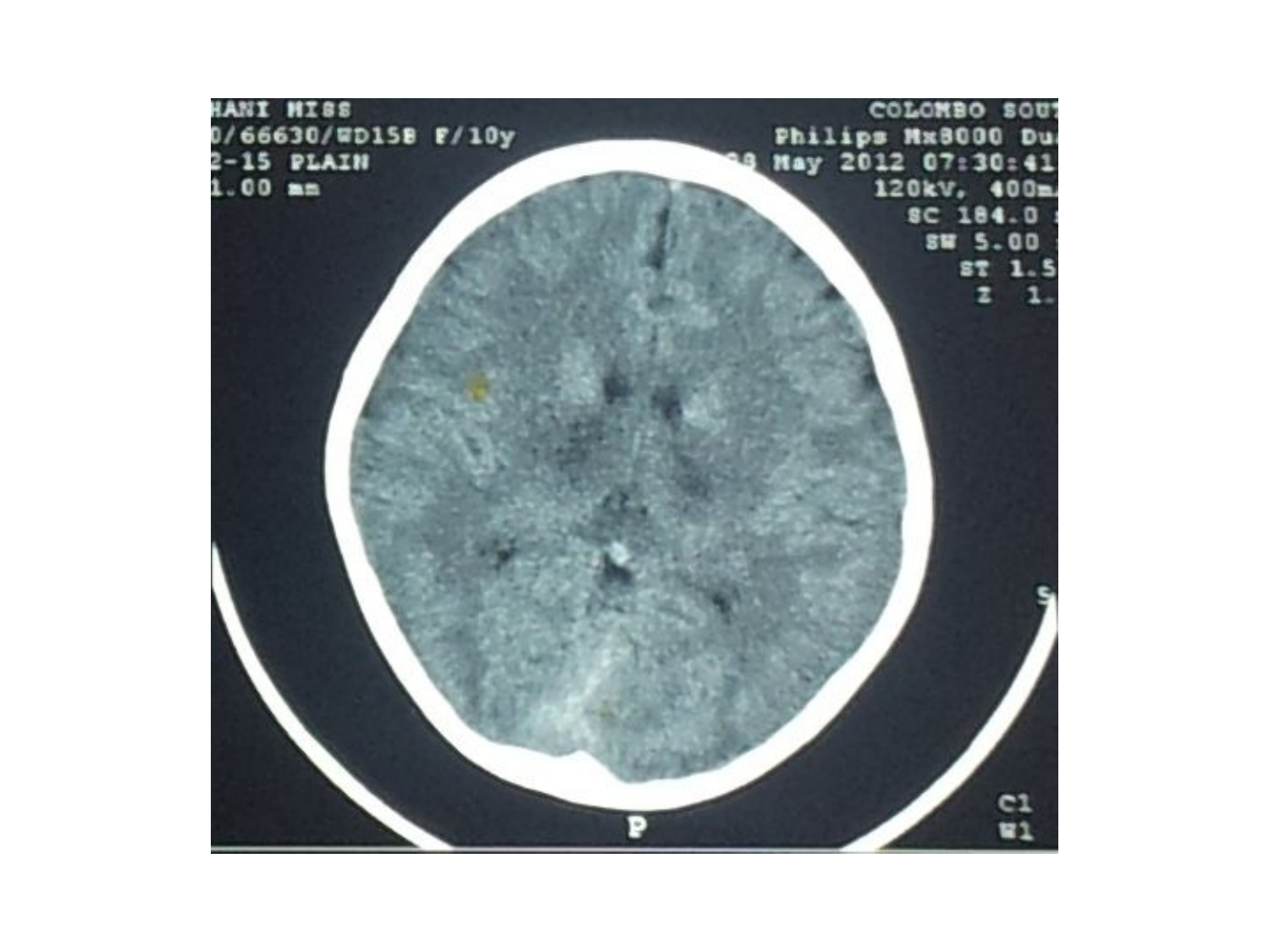

## Slide 4
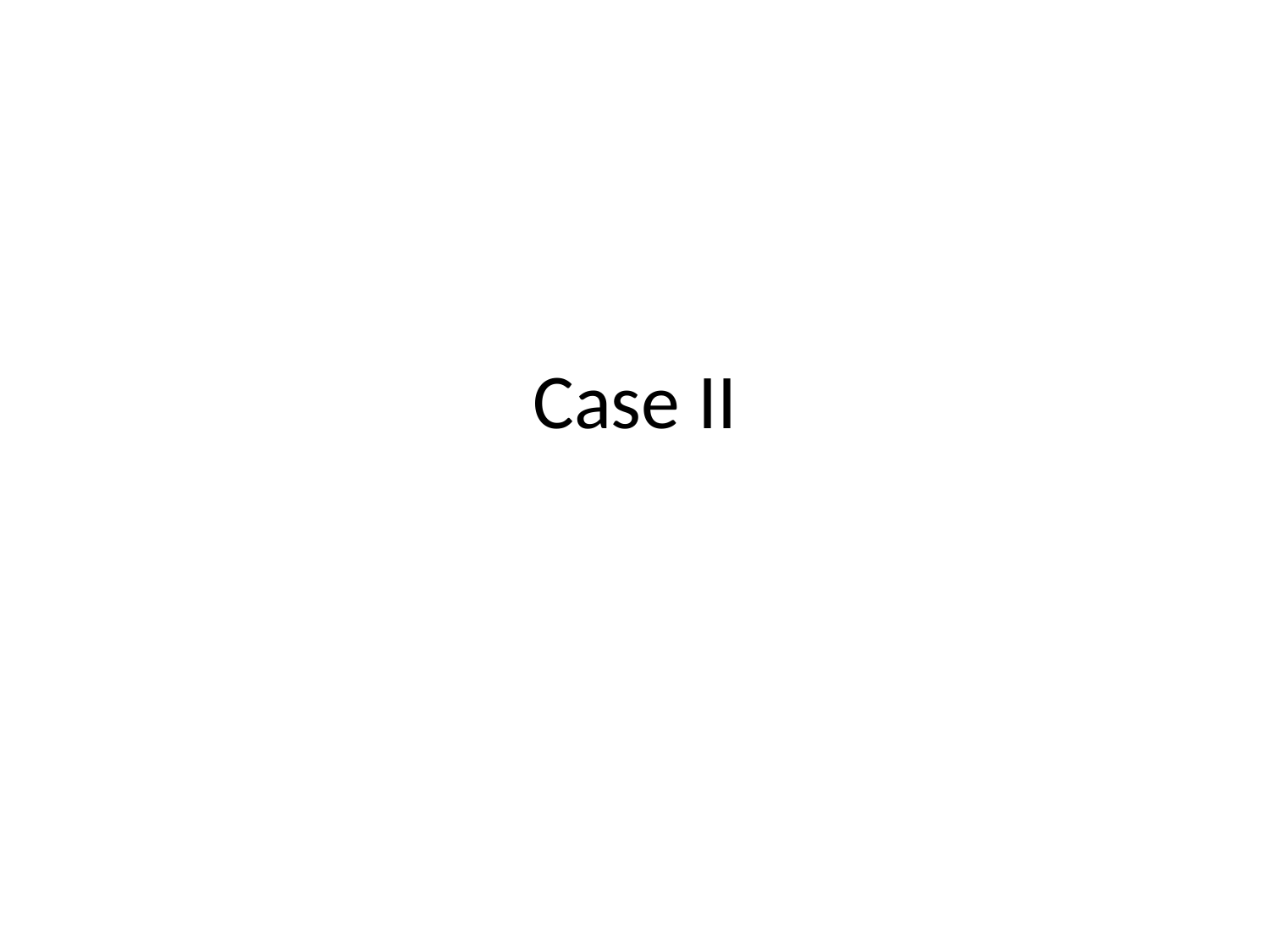

# Case II

## Slide 5
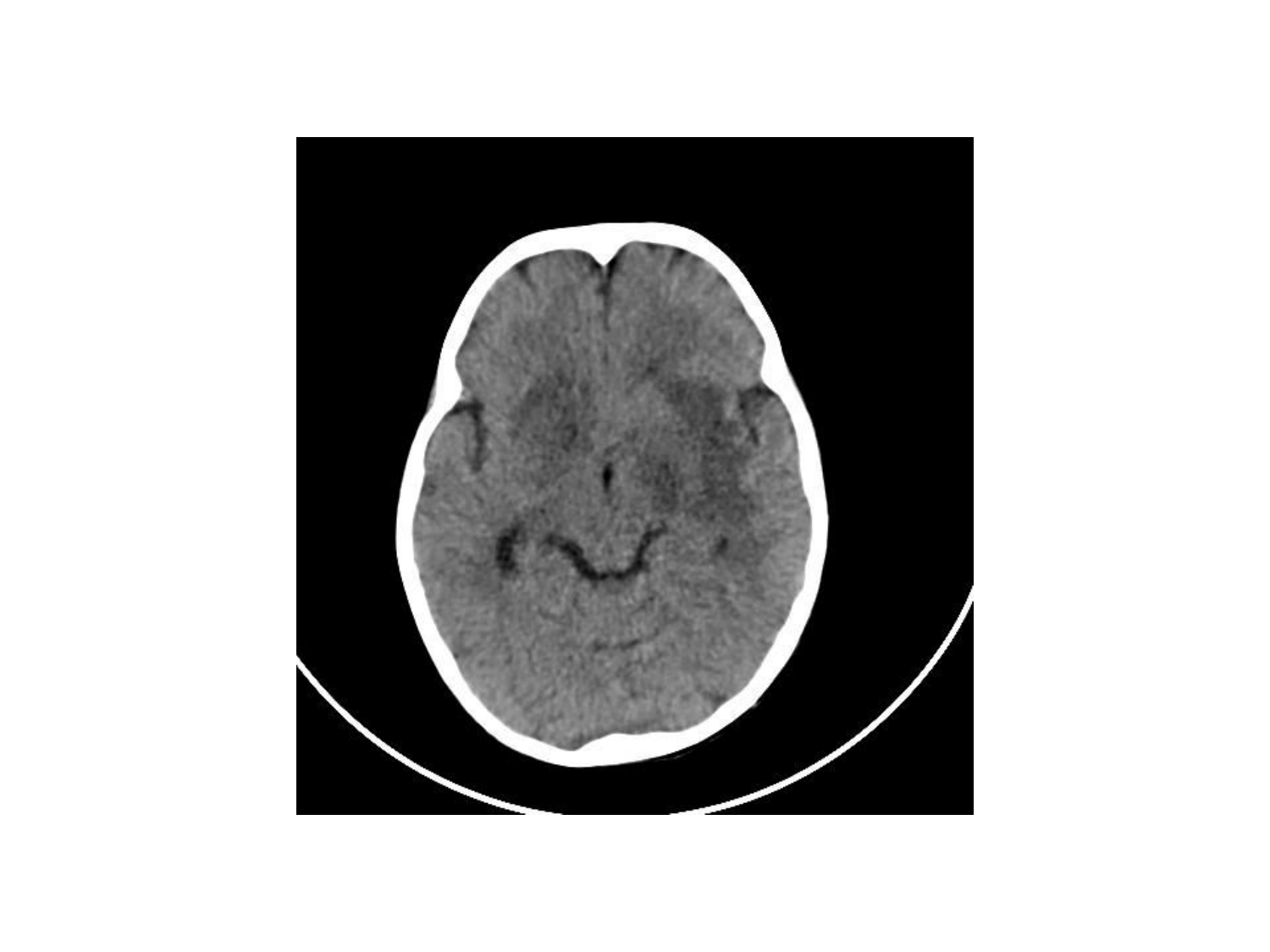

## Slide 6
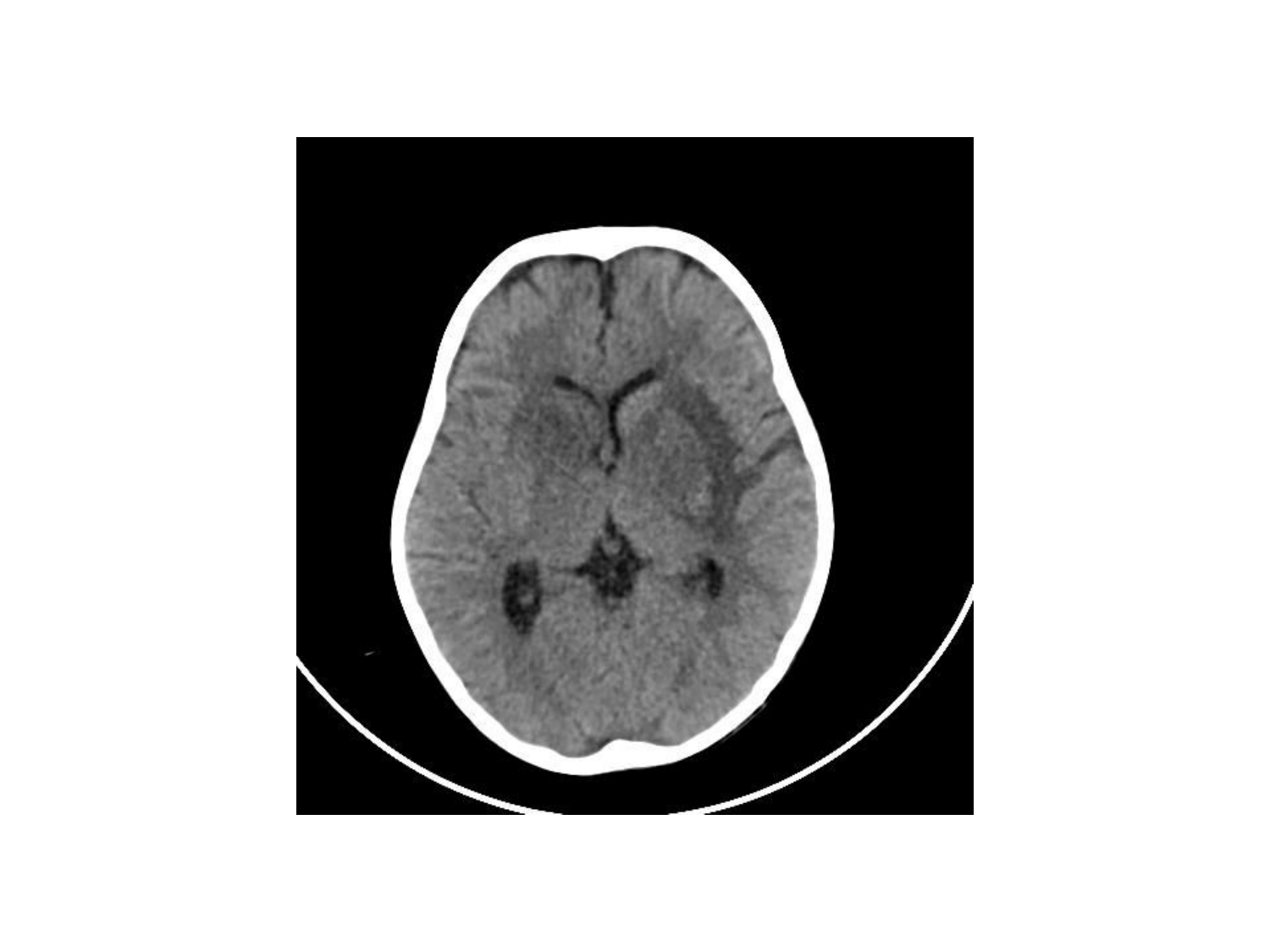

## Slide 7
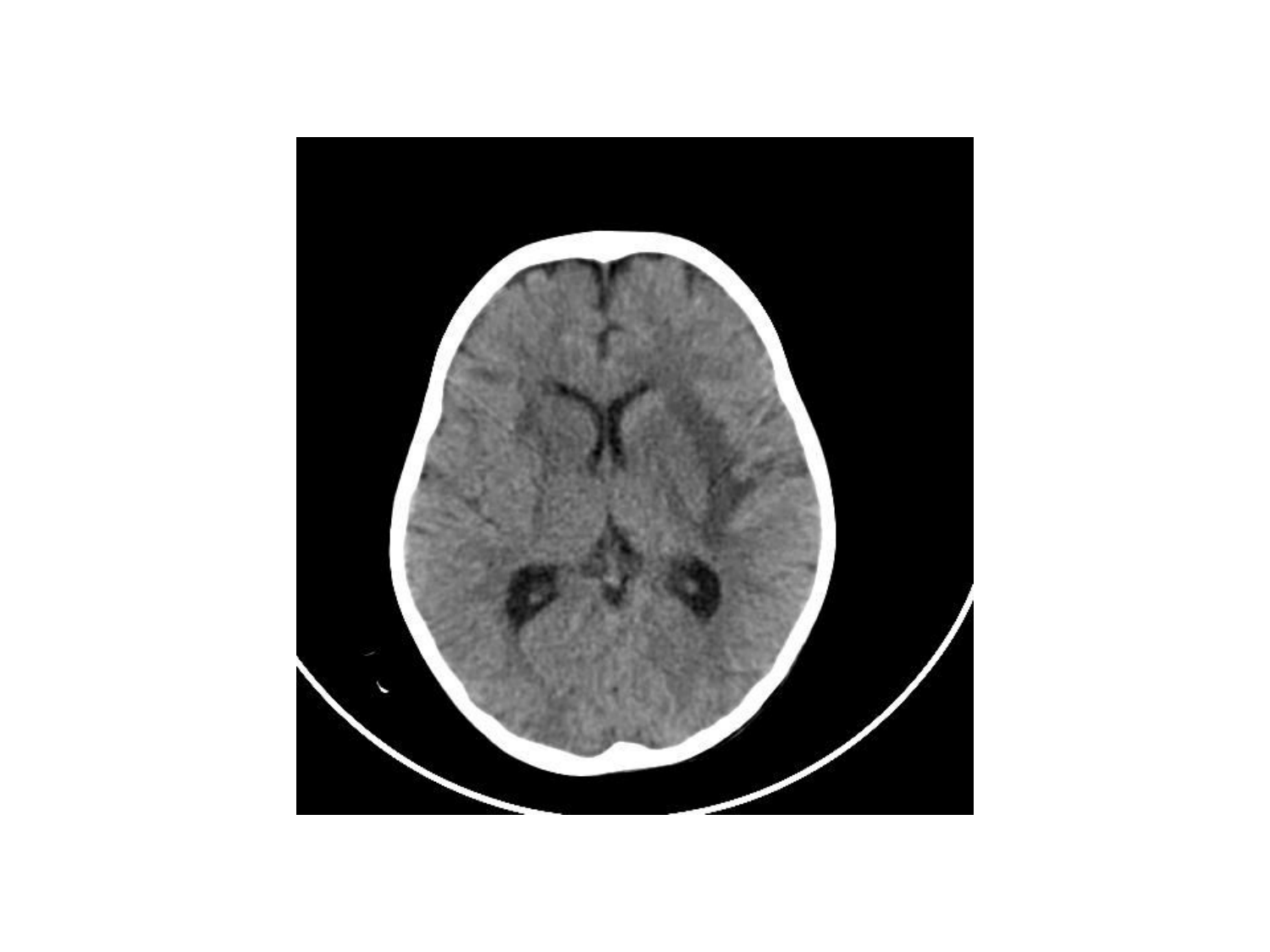

## Slide 8
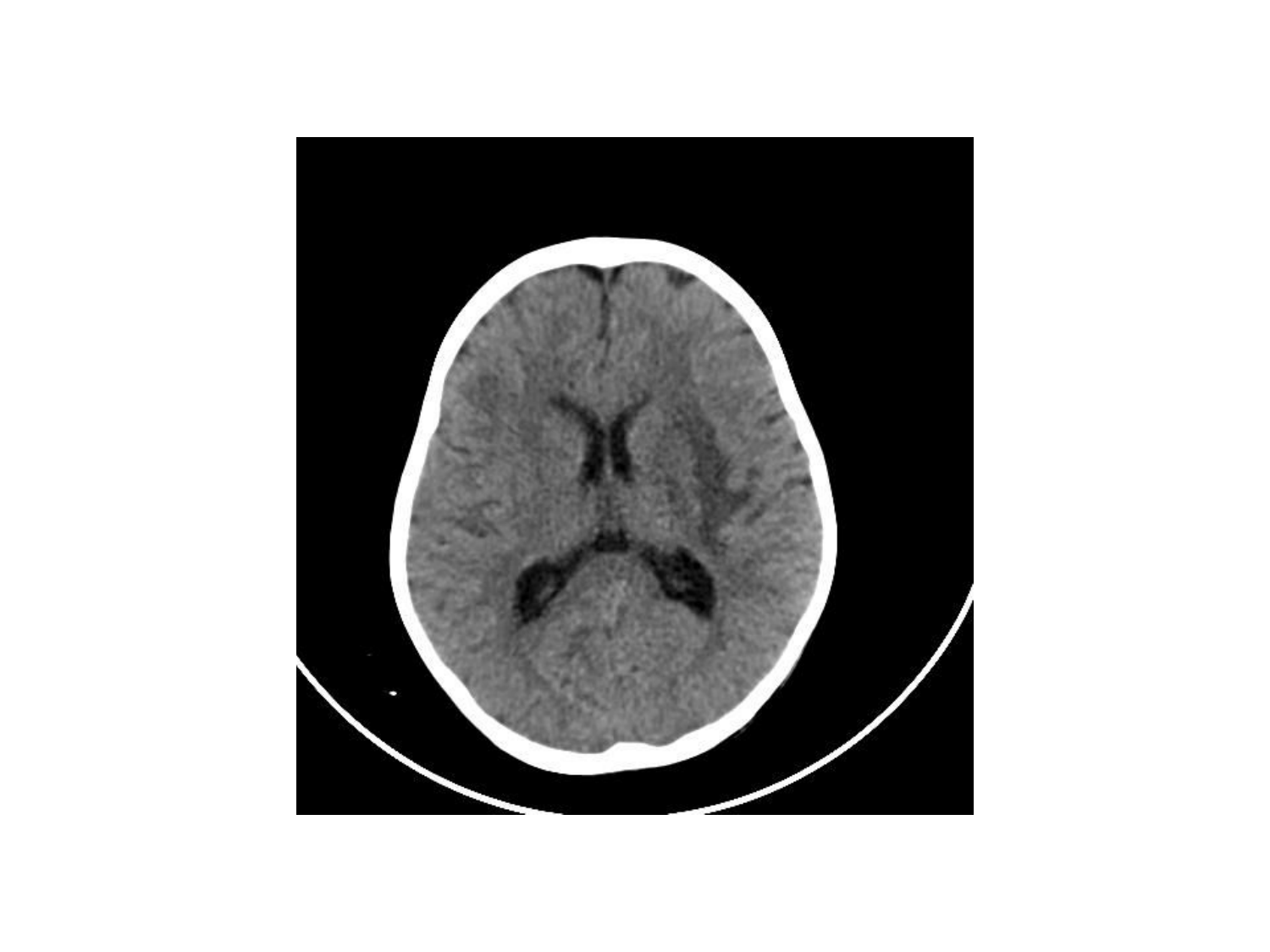

## Slide 9
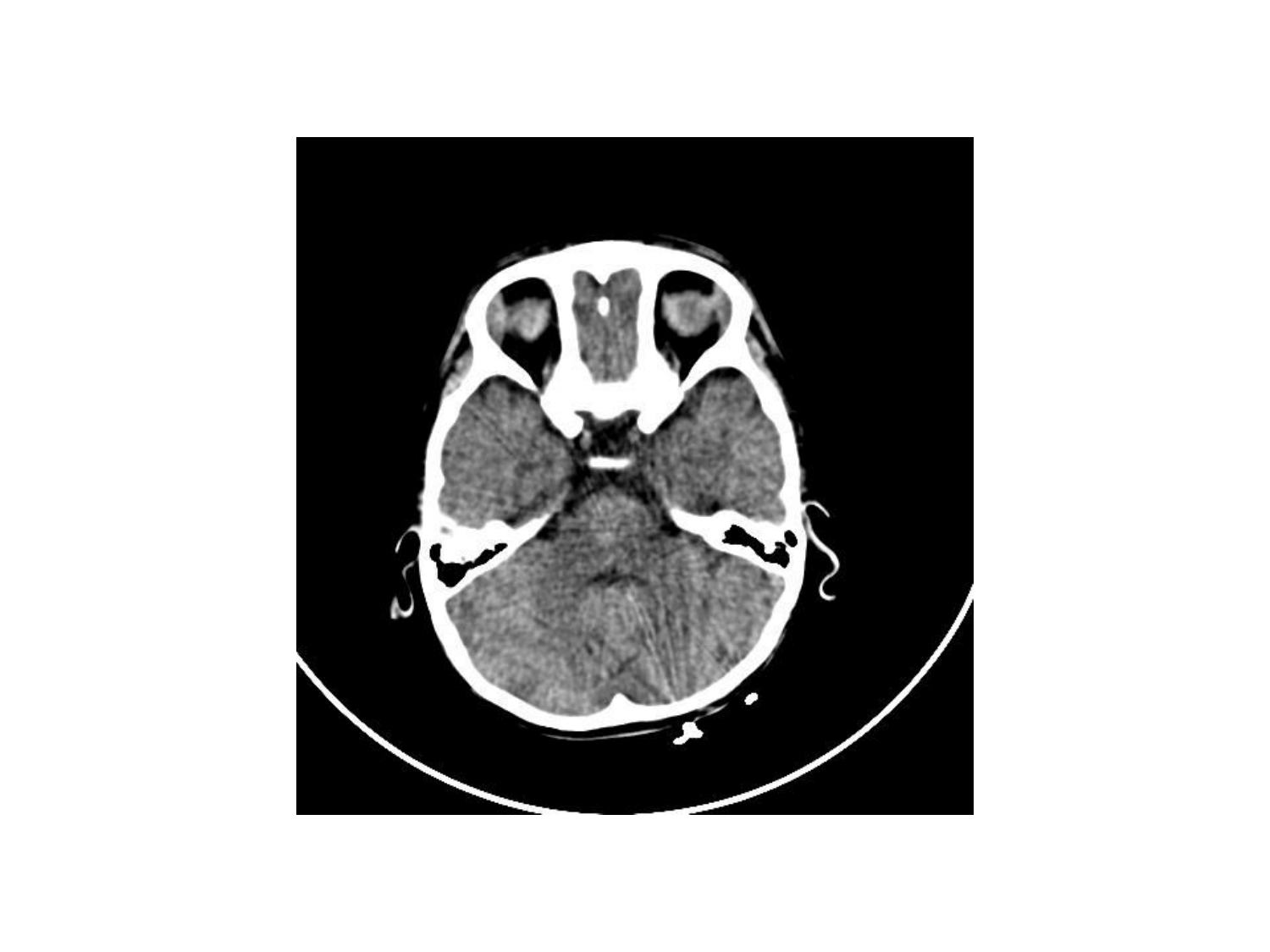

## Slide 10
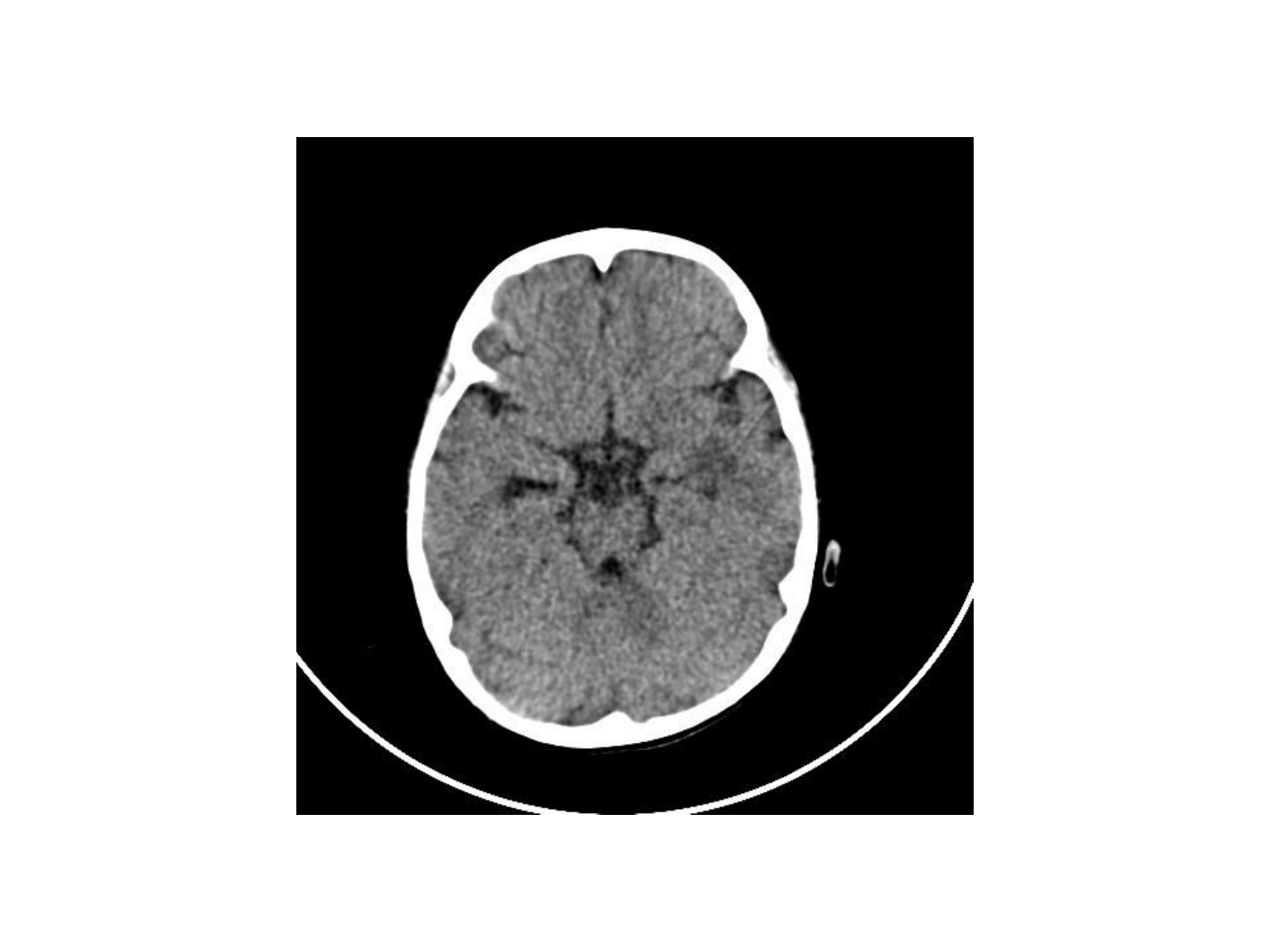

## Slide 11
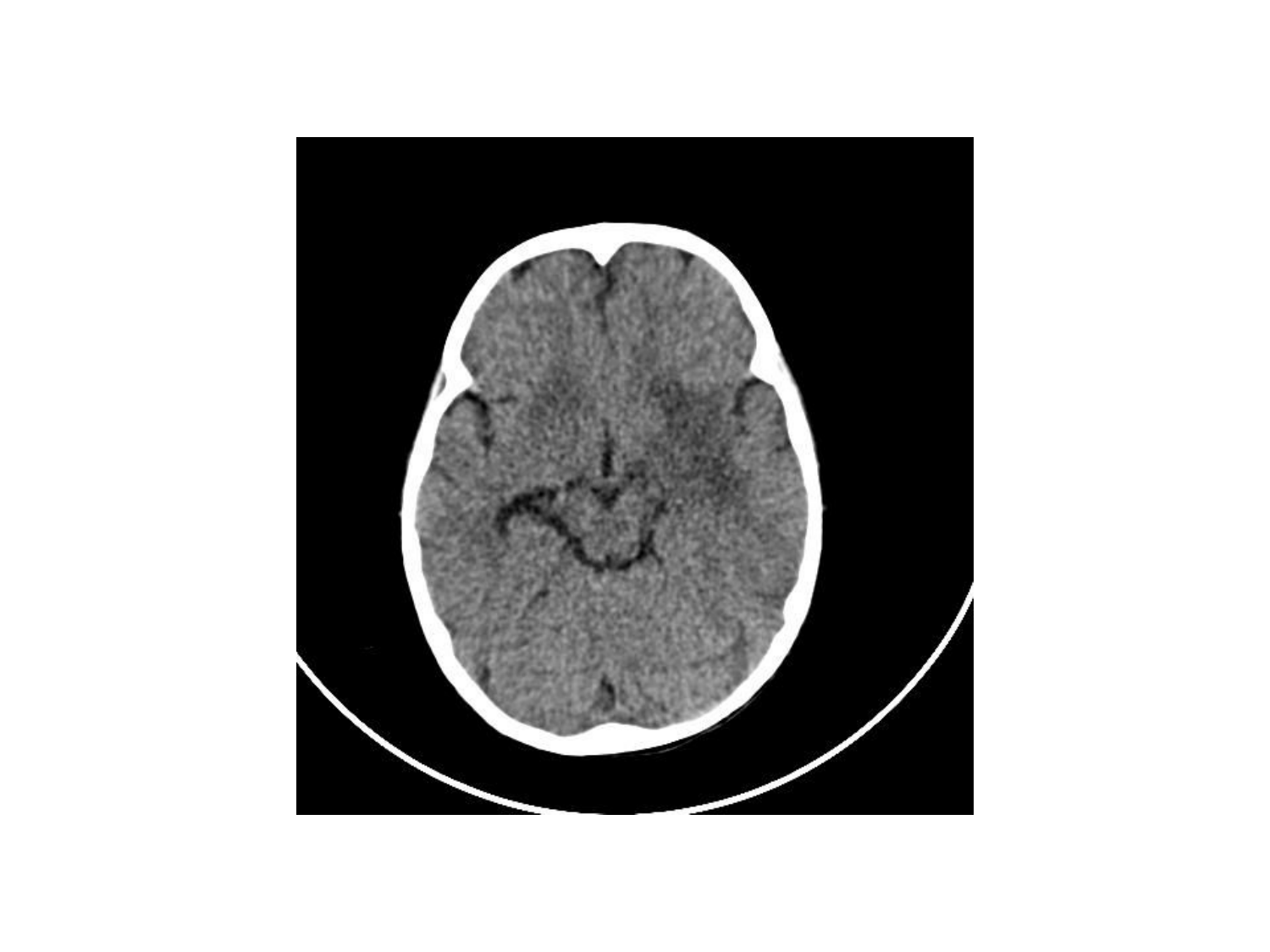

## Slide 12
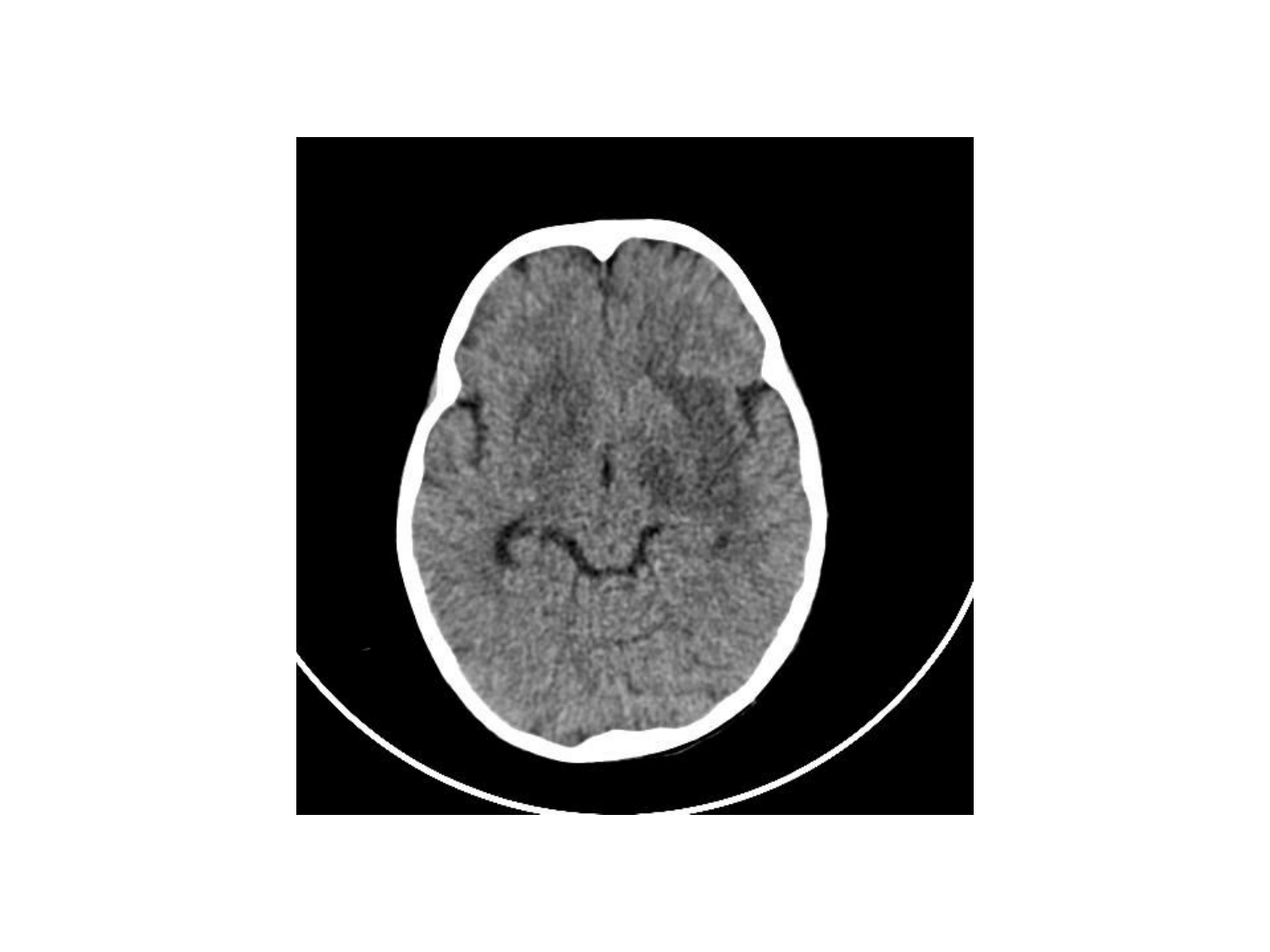

## Slide 13
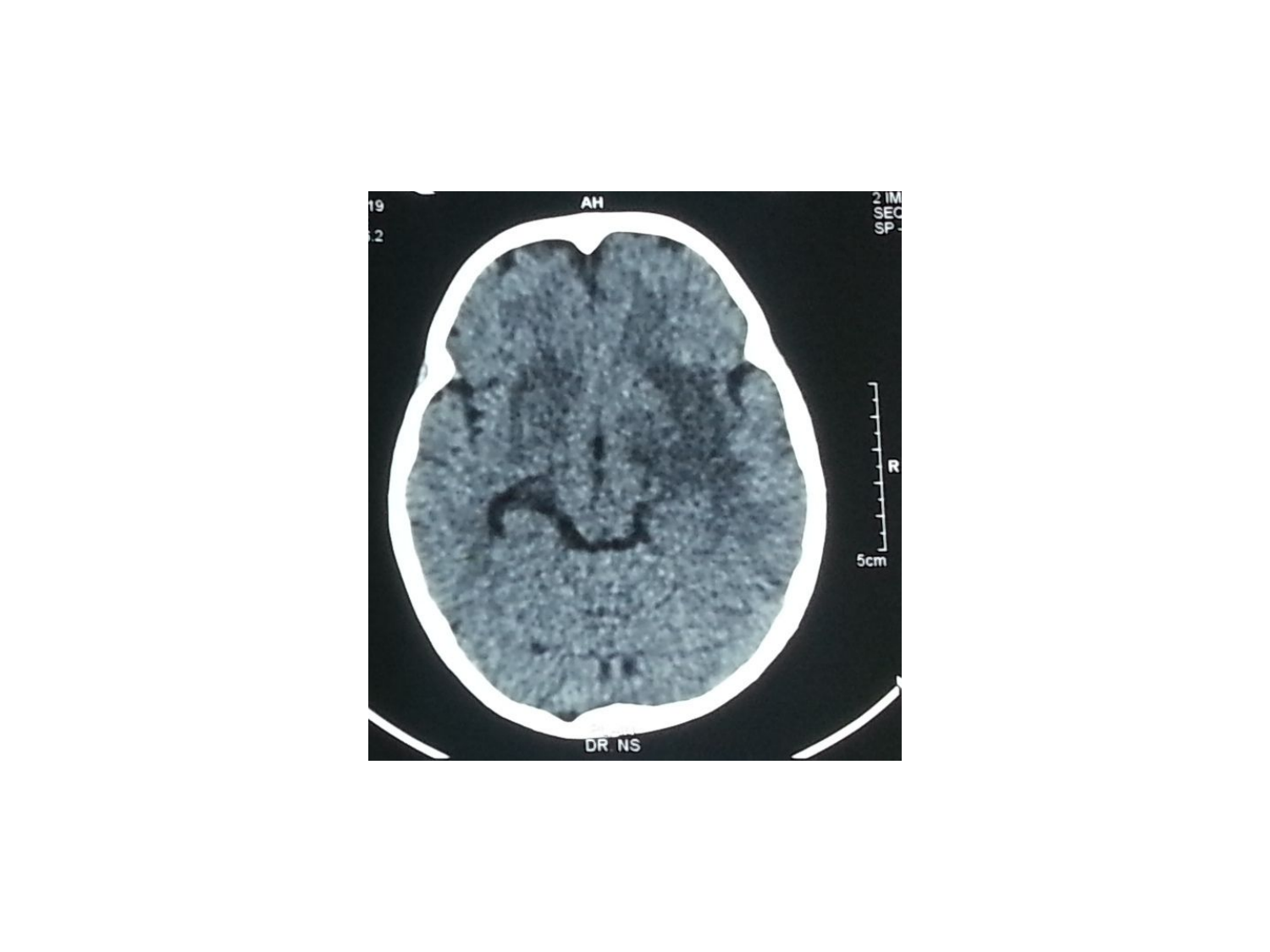

## Slide 14
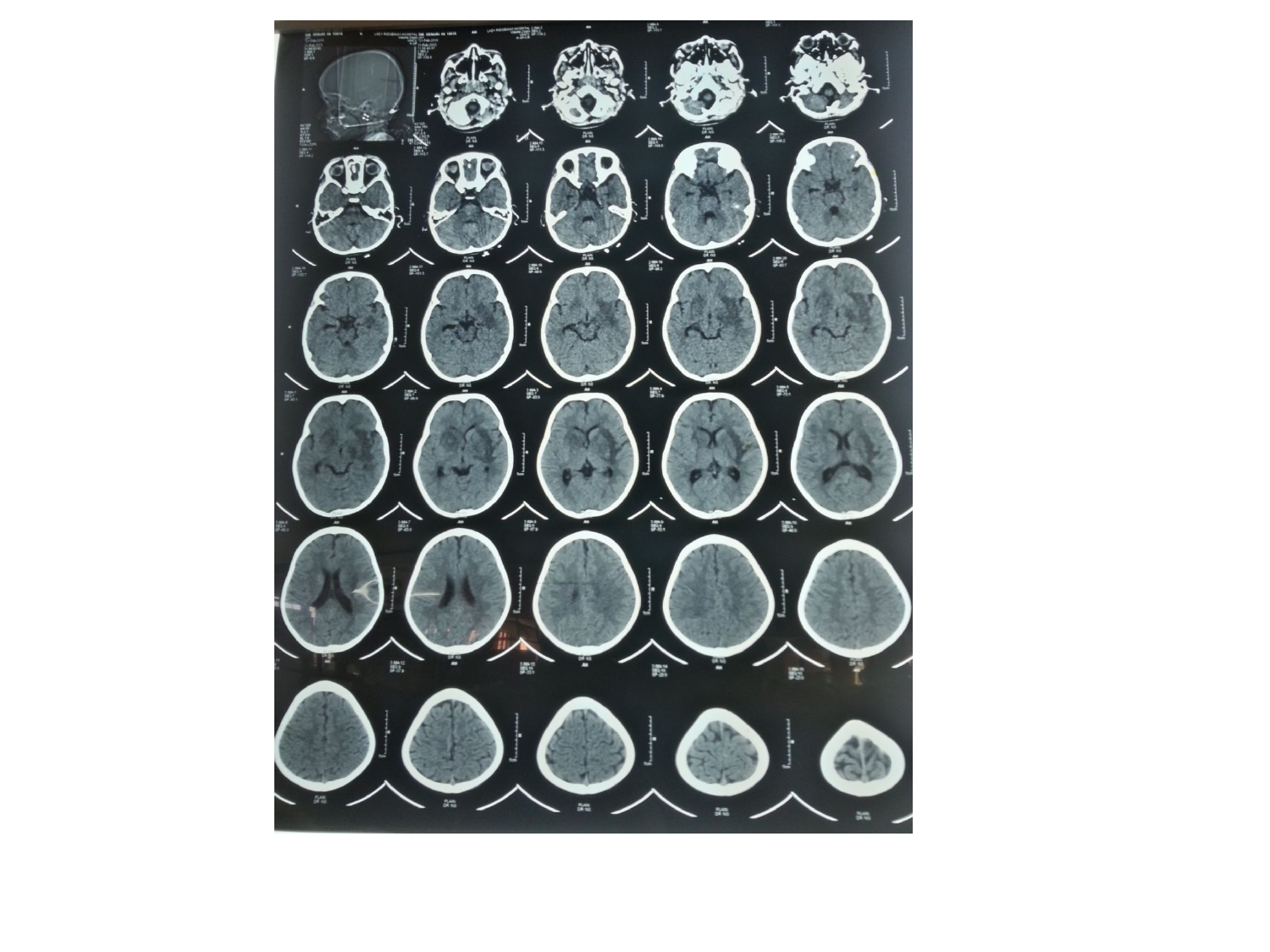

Supplement: Supplementary file 1 — Axial brain CT images showing hypodense areas in the region of thalamus and basal ganglia. [file 7961368.f1.pptx]
